# Cytosolic Delivery of Bioactive Cyclic Peptide Cargo by Spontaneous Membrane Translocating Peptides

**DOI:** 10.1021/acsomega.3c08701

**Published:** 2024-02-05

**Authors:** Ryan P. Ferrie, Taylor Fuselier, William C. Wimley

**Affiliations:** Department of Biochemistry and Molecular Biology, Tulane University School of Medicine, New Orleans, Louisiana 70112, United States

## Abstract

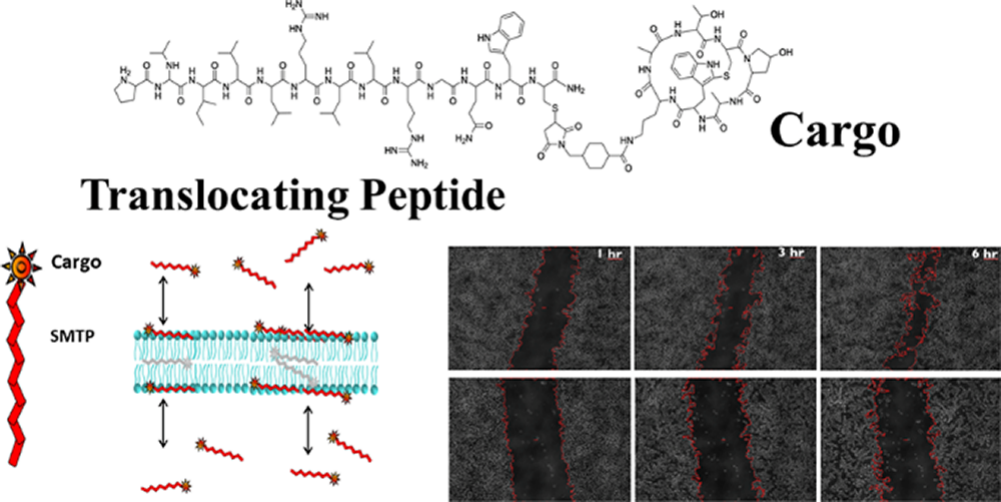

Cyclic peptides that inhibit protein–protein interactions have significant advantages over linear peptides and small molecules for modulating cellular signaling networks in cancer and other diseases. However, the permeability barrier of the plasma membrane remains a formidable obstacle to the development of cyclic peptides into applicable drugs. Here, we test the ability of a family of synthetically evolved spontaneous membrane translocating peptides (SMTPs) to deliver phalloidin, a representative bioactive cyclic peptide, to the cytosol of human cells in culture. Phalloidin does not enter cells spontaneously, but if delivered to the cytosol, it inhibits actin depolymerization. We thus use a wound-healing cell mobility assay to assess the biological activity of phalloidin conjugated to three SMTPs that we previously discovered. All three SMTPs can deliver phalloidin to the cell cytosol, and one does so at concentrations as low as 3 μM. Delivery occurs despite the fact that the SMTPs were originally selected based on membrane translocation with no cargo other than a small fluorescent dye. These results show that SMTPs are viable delivery vehicles for cyclic peptides, although their efficiency is moderate. Further, these results suggest that one additional generation of synthetic molecular evolution could be used to optimize SMTPs for the efficient delivery of any bioactive cyclic peptide into cells.

## Introduction

Cancer and other diseases result from aberrant signaling networks within cells,^1,2^ which are dominated by protein–protein interactions (PPIs). Classical small molecule drugs rarely succeed in targeting PPIs.^2^ However, peptides can readily interrupt PPIs with high selectivity and specificity.^1,2^ Peptides can target the broader, more extensive surfaces, typical of PPI interfaces, while small molecules favor protein binding sites within deep pockets, such as active sites in enzymes.^3^ Despite the suitability of peptides to act as PPI inhibitors, their delivery across the plasma membrane remains a major obstacle to the further development of peptide-PPI inhibiting drugs.^4−6^

Of particular interest is the development of cyclic peptides due to their biostability and ability to bind currently undruggable targets, especially PPI interfaces.^4,7^ Cyclic peptide drugs are structurally rigid and much more resistant to degradation than their linear counterparts.^8^ However, cyclic peptides remain largely membrane impermeant and are thus reliant on delivery vehicles for transport to the cytosol if they are designed to bind to intracellular targets.^5^ Two of the more common mechanisms utilized for the delivery of membrane-impermeable peptide drugs into cells are endocytic uptake and subsequent release/escape from endocytic compartments or direct translocation across the plasma membrane.^7^ Membrane-impermeable drugs conjugated to extracellular receptor ligands can activate receptor-mediated endocytosis. However, delivery by receptor-mediated endocytosis requires a minimal receptor density and sensitivity^9^ and, once uptaken, escape from the endocytic compartments is still required, as the default fate of the uptaken cargo is degradation or recycling. Conjugation of cargoes to cationic cell-penetrating peptides (CPPs) can enable some cytosolic delivery.^10−13^ While CPP-dependent delivery can occur either by active uptake or by concurrent pathways of direct translocation and active uptake,^10,14,15^ the latter is often dominant.^14^

Here, we test the application of spontaneous membrane translocating peptides (SMTPs) as suitable delivery vehicles for cyclic peptides. The family of SMTPs we test here was discovered by orthogonal high-throughput screening of a peptide library for members that spontaneously translocate across synthetic phosphatidylcholine membranes without membrane permeabilization.^16^ We subsequently showed that the most active SMTPs, called TP1, TP2, and TP3, translocate directly across the plasma membrane of eukaryotic cells, bypassing active uptake, to directly and efficiently deliver the membrane-impermeant dye tetramethylrhodamine (TAMRA) to the cell cytosol.^16,17^ In vivo, the SMTPs enable efficient delivery of the TAMRA cargo to many tissues.^17^ Unlike classical CPPs, such as tat and penetratin,^14^ SMTPs act as monomers, do not form complexes or pores, and do not cause membrane permeabilization.^17,18^ SMTPs deliver membrane-impermeant small molecule cargoes via direct translocation.^16−18^ Here, we test the ability of SMTPs to deliver a bioactive cyclic peptide cargo to the cell cytosol, an activity that has not been previously tested.

The cyclic peptide that we used as a model cargo for small (<1 kDa), cyclic peptide drug candidates was the phallotoxin phalloidin, produced by the Death Cap mushroom *Amanita phalloides*. Phalloidin is a 780 Da bicyclic heptapeptide that binds to and stabilizes filamentous actin, preventing actin depolymerization and subsequent cellular division and chemotaxis.^19^ Phalloidin itself is not membrane permeable, but can bind to the actin of live cells if delivered across the plasma membrane into the cytosol.^20^ Dye-labeled phalloidin is widely used for fluorescent imaging in fixed, permeabilized cells due to its high affinity and specificity for F-actin,^21^ but it does not spontaneously enter most live cells. While phalloidin itself is a nontherapeutic agent, here, it represents the broad class of small, cyclic peptides that bind to cytosolic proteins. As such, the successful delivery of phalloidin promotes the idea that optimized SMTPs may be useful vehicles for the delivery of other small cyclic peptides.

We hypothesized in this work that phalloidin, covalently conjugated to SMTPs, can be delivered directly to the cytosol of human cells and retain its biological activity. We took advantage of the fact that delivery of phalloidin inhibits cell migration,^20,22,23^ which was used as our primary metric in determining the successful delivery by SMTPs. Specifically, scratch or wound-healing assays were used to assess phalloidin delivery by measuring cell migration.^24^ In this work, we have, for the first time, expanded the application of SMTPs to demonstrate that they can deliver cyclic bioactive peptides, an important class of potential drugs, to the cytosol of live cells.

## Methods

### Peptide Synthesis, Conjugation, and Purification

d-Amino acid versions of TP1 (plillrllrgqwc-amide, mw = 1579.9 g/mol), TP2 (pliylrllrgqwc-amide, mw = 1629.9 g/mol), and TP3 (rrillqllrgqwc-amide, mw = 1653.9 g/mol) were synthesized via solid phase peptide synthesis (SPPS) on Tentagel MB (loading capacity = 0.23 mmol/g) using previously established methods.^11,16,25−28^ In brief, a photolabile linker (Creosalus, RT1095) was coupled to the beads, followed by d-isoform amino acids for each peptide from the C- to N-terminus. All amino acid additions were performed in a cyclic and stepwise fashion, wherein FMOC-protecting groups were cleaved from the N-terminus of the previous amino acid by 30/70 (v/v) piperidine/DMF, and the next amino acid was conjugated at a 2-fold molar excess using standard FMOC chemistry. If coupling was unsuccessful after three attempts, beads were capped with a solution of 2% DIPEA, 2% acetic anhydride, and 96% DMF (% v/v). All synthesis reactions occurred in peptide synthesis vessels with fritted disks at room temperature (RT), while rocking; beads were washed five times with DMF between all reactions. Coupling was monitored visually via the Kaiser test.

**Figure 1:**
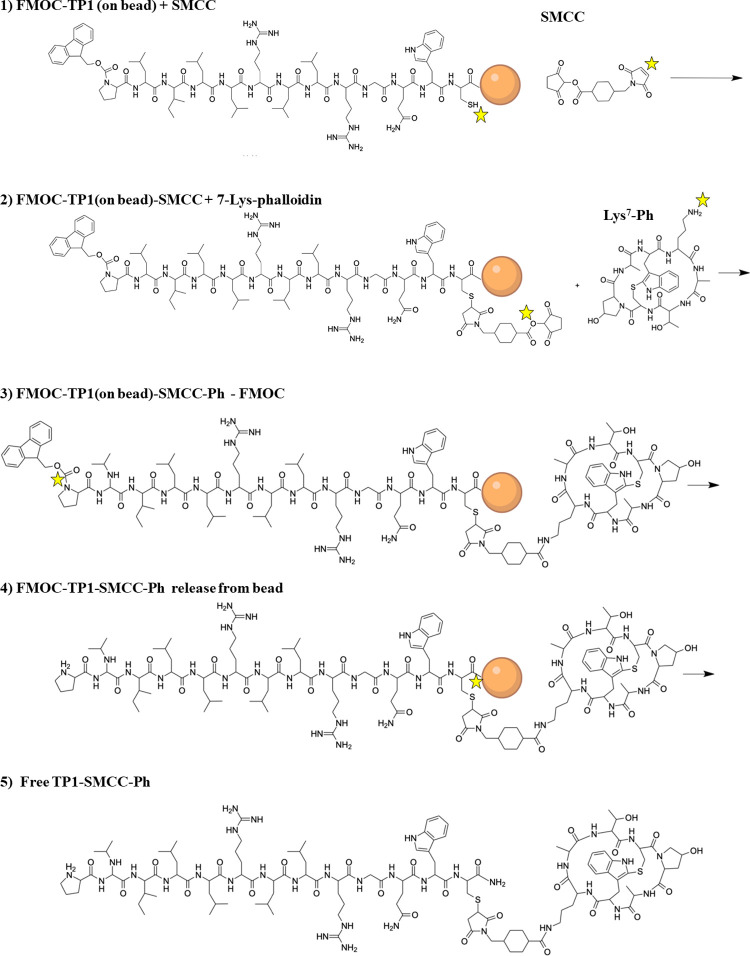
Solid phase synthesis scheme for SMTP-phalloidin conjugates. Step 1: SMTP peptides were synthesized manually on Tentagel-S-NH_2_ solid phase synthesis resin after the addition of a UV-cleavable photolinker. Side chain protecting groups were removed with trifluoroacetic acid, and the N-terminal FMOC-protecting group was not removed. The SMCC bifunctional cross-linker was coupled to the peptide C-terminal cysteine residue via its maleimide group. Step 2: 7-lys-phalloidin was conjugated to the peptide-SMCC conjugate on beads. Step 3: The FMOC group was removed from the amino terminus of the peptide. Step 4: The SMTP-SMCC-Ph peptide amides were released from the resin with 5 h of UV light at 350 nm with continuous mixing. Step 5: The free SMTP-SMCC-Ph conjugates in pure form were released from the beads by UV light and dissolved in DMSO at 1–2 mM total concentration.

Following completion of synthesis, side chain protecting groups were cleaved with Reagent B (88% TFA, 5% H_2_O, 5% phenol, 2% TIPS), but FMOC was not removed from the amino terminal group and the peptide was not released from the beads. TP1, 2, and 3 were then conjugated, on the beads, to the phalloidin cargo as follows: a 5-fold molar excess of the heterobifunctional cross-linker SMCC was added to beads in DMF to enable the maleimide-cysteine reaction to take place. This reaction was incubated for 2 h, and the beads were washed. Following washing, Lys7-phalloidin (Bachem) in DMF containing 1% DIPEA was added to beads at a molar ratio of 1:2 (peptide:Ph) and incubated at room temperature overnight. This reaction couples phalloidin to peptide-SMCC via the succinimidyl ester moiety. Lastly, the FMOC group was removed with 30% piperidine in DMF, and the beads were extensively washed. UV light-induced cleavage in hexafluoroisopropyl alcohol (HFIP) was used to release the TPx-S-Ph conjugates from the beads. HFIP-containing peptide was extracted from beads and dried under vacuum, and the peptides were resuspended at 1–5 mM in DMSO for cell culture experiments. Each step in the on-bead conjugate synthesis (see Figure 1) was pushed to completion using stoichiometric excesses of soluble reagents compared to bead-tethered peptides, followed by extensive washing of the bead-tethered product. In this way, the final SMTP-SMCC-phalloidin conjugates released from the beads had the expected mass and were essentially pure, as shown by the mass spectrometry data in Supplementary Figures S1 and S2. Peptide concentrations were determined using UV–vis spectrometry.

It is important to note that maleimide-thioether bonds, including the SMTP-SMCC bond are not infinitely stable in intracellular environments because they undergo retro-Micheal thiol exchange reactions.^29,30^ The 1–10 mM reduced glutathione present in a typical mammalian cell cytosol is the most abundant free thiol for exchange. However, reported exchange rates for methyl-cyclohexyl-maleimides (such as SMCC) are on the order of 400 h,^29^ whereas our critical wound closure experiments are from 1 to 8 h. Further, our wound closure experiments show no deviation from linearity when we compare 1–8 h suggesting that no changes in peptide-SMCC-phalloidin concentration/activity are occurring during this time period. Lastly, if the retro-Micheal thiol exchange is unexpectedly occurring on these short time scales, it would give rise to a glutathione-SMCC-phalloidin conjugate, which would likely retain activity; we show later that the peptide attached to phalloidin at the seventh position via SMCC does not significantly affect the bioactivity of phalloidin.

The synthesis of the SMTP peptides was verified by HPLC and mass spectrometry, and the identity and purity of the SMTP-SMCC-Ph conjugates and intermediates were validated by MALDI mass spectrometry. See Supplementary Figures S1 and S2 for quality control mass spec data. HPLC was done on a Shimadzu Prominence system, using a Machery-Nagel C18 reverse phase column at 35 °C developed in water acetonitrile with 0.1% v/v TFA. Peptide was detected by UV absorbance at 220 and 280 nm and fluorescence detection at excitation 280 nm and emission 340 nm. After synthesis and release of the final conjugate, reversed-phase HPLC showed a single peptide peak with ≥95% purity. MALDI MS was performed with a Bruker Autoflex3 Matrix assisted laser desorption ionization-time of flight mass spectrometer. The masses of peptides alone, of intermediate products, and of the final SMTP-SMCC-phalloidin conjugates were as expected. See Supplementary Figures S1 and S2 for MALDI mass spectrometry data.

### Actin Depolymerization

An actin polymerization biochemistry kit (Cytoskeleton, Inc.) was used to assay the bioactivity of the phalloidin conjugates. All experiments were performed in a 96-well format and read on a fluorescent plate reader (Biotek Synergy II). Actin-pyrene monomers formed filamentous actin by using the supplied polymerization buffer, which caused pyrene excimer fluorescence (excitation 365 nm and emission 407 nm). Serial dilutions of phalloidin or TP2-SMCC-Ph were added to the filamentous actin-pyrene as well as a no peptide control. A supplied depolymerization buffer was then added to samples, and loss of fluorescence was monitored on the plate reader as a function of time. Rates of depolymerization were plotted for each sample by performing linear regression on the linear portion of the kinetic curves. Calculated rates are normalized to the buffer-only controls.

### Cell Culture

Validated HeLa human ovarian cancer cells and HT1080 human epithelial fibrosarcoma cells were obtained from ATCC and were used at a low passage number without modification. All cells were maintained in T75 flasks in an incubator at 37 °C, 5% CO_2_ in complete media: DMEM containing 10% (%v/v) FBS, glucose, sodium pyruvate, l-glutamine, nonessential amino acids, and antibiotics. Serum-free media (SFM) was prepared in the same fashion as complete media but lacked FBS. Cells were maintained daily with two DPBS washes and replacement of complete media. When ≥90% confluent, cells were passaged using trypsin and seeded into another T75 flask and/or 96-well plates.

### Cell Migration Assay

A total of 30,000 HT1080 cells were seeded into each well of a 96-well plate in complete media and incubated for 24 h. Cells were washed with DPBS and incubated in serum-free media (SFM) for the next 48 h. Treatment with TP1/2/3-S-Ph, cytochalasin D, or media control, containing DMSO equal to treatment (% v/v), was done for four h at 37 °C. Cells were washed three times with DPBS and then scratched with 10 μL pipet tips on a multichannel pipettor. Evaluation of area closure was performed by phase contrast microscopic imaging of each well at 4× magnification, hourly, for 24 h with the Biotek Cytation 5 cell imaging reader and the BioSpa 8 automated incubator. Only the linear portion of the time course, up to 8 h, was used for analysis. For each condition studied, the scratch assay was performed in at least 6 biologically independent experiments, and each experiment consisted of 2–3 technical replicates.

### Image Quantification

ImageJ was used to trace the perimeter of each scratch. The gap area (GA) of each scratch was generated with ImageJ via batch processing with a lower threshold of 0 and an upper threshold of 60. The gap area for treatment groups was averaged and relativized as follows:
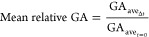


Mean relative GA was plotted over time, and the absolute value of the slope from 1 to 8 h for each treatment was determined. Lastly, slopes were normalized against SFM controls. Wells were excluded that did not have a cell border on both sides of the scratch and/or contained bubbles that obscured the image to the extent that it was untraceable by ImageJ.

## Results

For this work, we developed a facile method of on-bead conjugation of the phalloidin cargo to the SMTP peptides using the heterobifunctional cross-linker SMCC, Figure 1. The synthetic scheme is shown in Figure 1. Briefly, the peptides were manually synthesized on Tentagel-S-NH_2_ solid phase synthesis resin, attached to the resin by a UV-cleavable photolabile linker. Side chain deprotection with trifluoroacetic acid plus scavengers was carried out, while the amino terminus remained blocked by an FMOC group. The resin-bound peptide was reacted with a 5-fold excess of SMCC in dimethylformamide (DMF) to couple SMCC to the C-terminal cysteine residue by a maleimide-dependent thioether linkage. After excess reagents were washed from the resin, 7-lys-phalloidin was added in DMF with 1% (v/v) diisopropylethylamine (DIPEA) to couple the cargo to the peptide-SMCC conjugate via a succinimidyl-catalyzed amide bond formation. After washing excess reagents, the N-terminal FMOC group was removed with 30% piperidine in DMF, and the peptide-SMCC-Ph-amide conjugate was released from the resin by 5 h of UV light at 350 nm with constant mixing.

**Figure 2:**
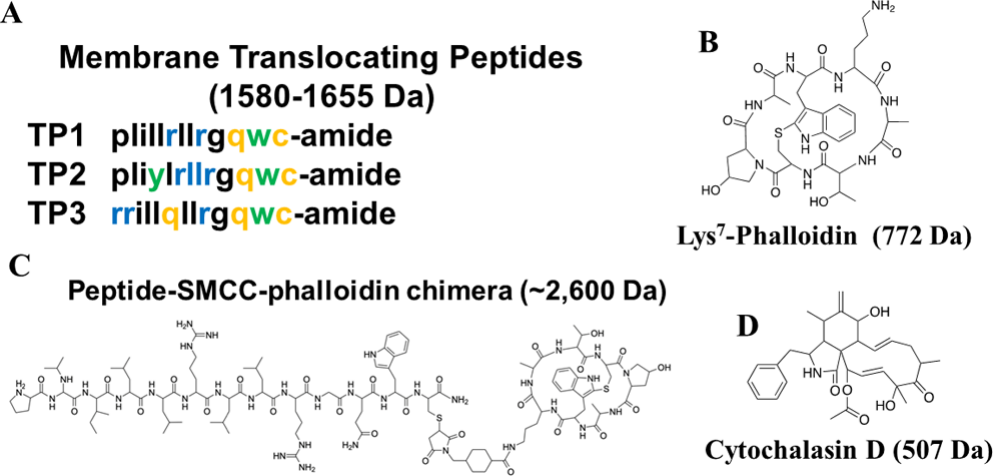
Peptides, cargos, and conjugates. (A) Sequences of the three SMTPs used in this work. In this work, we used d-amino acid variants for stability. (B) Chemical structure of the test cargo, phalloidin, a bicyclic heptapeptide that inhibits f-actin depolymerization. (C) Structure of TP2 with phalloidin attached by the heterobifunctional cross-linker, SMCC. (D) Chemical structure of cytochalasin D, a membrane-permeant small molecule inhibitor of actin, that we used as a positive control.

Our goal in this work was to test the ability of the membrane translocating peptides TP1, TP2, and TP3 to deliver phalloidin to the cytosol of living cells. The sequences of the SMTPs TP1, TP2, and TP3 are shown in Figure 2A. The heterobifunctional linker, SMCC, was used to covalently conjugate Lys7-phalloidin, Figure 2B, to the SMTPs, resulting in the chimeras TP1-S-Ph, TP2-S-Ph, and TP3-S-Ph. The chimeric structure of TP2-S-Ph is shown in Figure 2C as an example. The small molecule actin-inhibiting drug cytochalasin D, Figure 2D, was used as a control.

**Figure 3:**
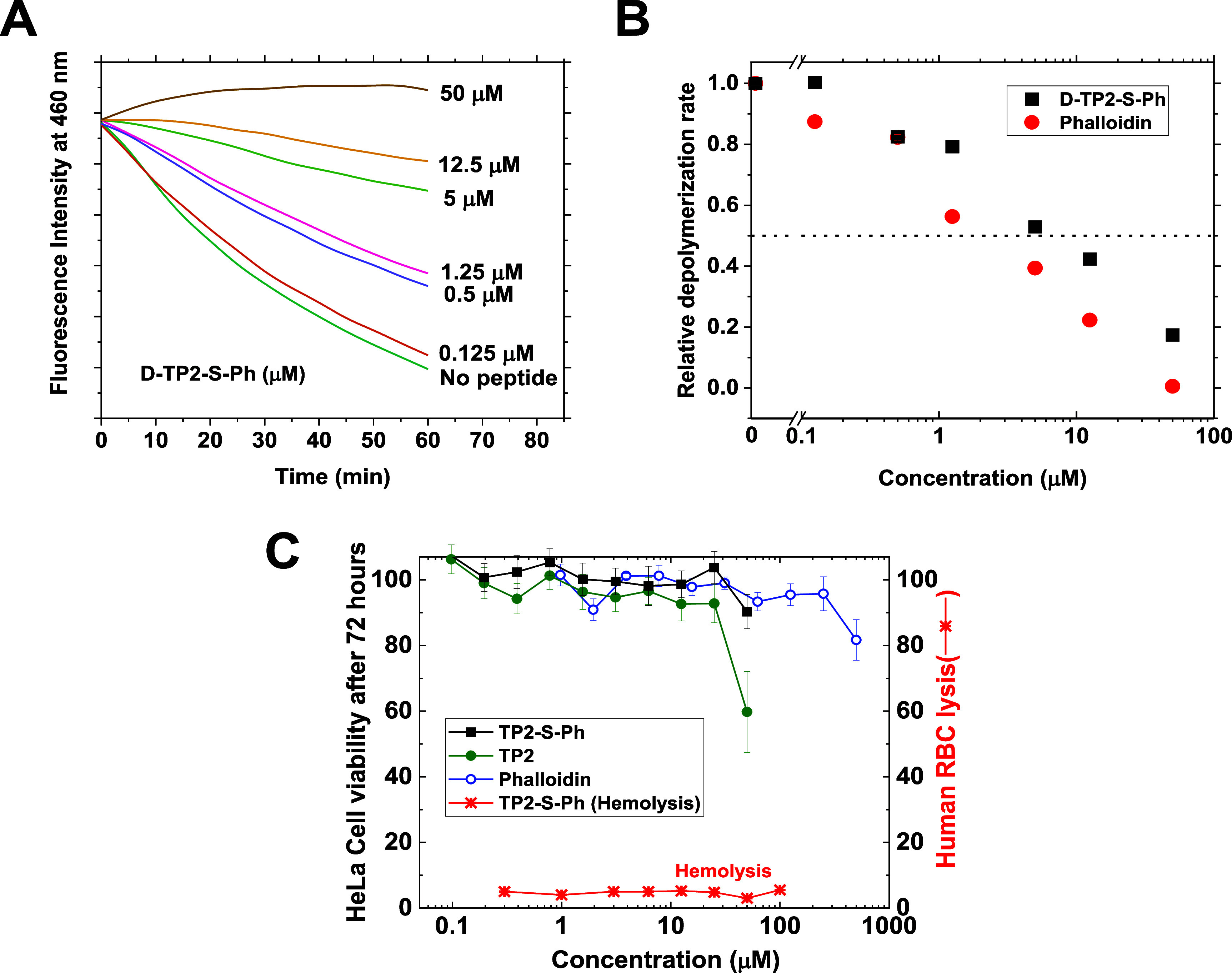
Control experiments. (A) Example *in vitro* actin depolymerization assay. Depolymerization of pyrene-labeled actin from Cytoskeleton, Inc. was initiated at time zero in the presence of 0–50 μM TP2-S-Ph conjugate. Pyrene excimer fluorescence was excited at 390 nm and monitored at 460 nm over 1 h. (B) Initial depolymerization rates from experiments such as the one shown in Panel A were measured by linear regression and averaged. We show depolymerization rates versus concentration for phalloidin and for the D-TP2-S-Ph conjugate. EC_50_ values are ∼3 μM for phalloidin and ∼6 μM for D-TP2-S-Ph. (C) Cytotoxicity and hemolysis caused by TP2, phalloidin, and the TP2-S-Ph chimera. Left axis: HeLa cells were incubated with the indicated concentration of compound for 4 h, and cell viability was measured by Alamar Blue fluorescence after 72 h. Right axis: washed human red blood cells were incubated for 1 h, and hemoglobin release was measured.

The 7-position of natural phalloidin is a 4,5-dihydroxy leucine residue. However, the fact that dye-labeled 7-Lys phalloidin is widely used as a microscopy stain for actin demonstrates that the 7-position can be modified, including large polar groups, without fundamentally altering the functionality of phalloidin. Here, we use the amino group of 7-Lys of phalloidin to couple phalloidin to the SMTPs using the cross-linker SMCC. To verify that this conjugation strategy does not block the function of phalloidin, we used *in vitro* actin depolymerization assays to show that the SMTP-SMCC-phalloidin conjugates effectively inhibit actin depolymerization. In Figure 3A, we show example kinetics curves for depolymerization of pyrene-labeled actin. Pyrene excimer fluorescence is measured such that depolymerization will drive a decrease in intensity. Polymerized actin, with added phalloidin or TP2-SMCC-Ph, is triggered to depolymerize at time zero, and the rate of excimer decrease is measured. In Figure 3B, the concentration dependence of depolymerization rates shows that both natural phalloidin and TP2-SMCC-Ph inhibit actin depolymerization with EC_50_ values < 5 μM.

The SMTPs have been shown to have low toxicity and low cell lysis activity.^16,17^ To further test for indirect cytotoxicity, we incubated HeLa cells with TP2-S-Ph, TP2 alone, and free phalloidin for 72 h, Figure 3C, and measured cell viability using Alamar Blue. Neither the conjugated TP2 nor TP2 alone showed any toxicity in the 0–25 μM concentration range studied here. TP2 alone showed some toxicity at 50 μM, but the conjugate did not. Phalloidin alone, up to 1 mM, showed no cytotoxic effects, which verifies the expectation that it does not enter cells spontaneously. We also measured hemolysis of red blood cells by TP2-S-Ph and found no hemolytic activity.

To measure the delivery of phalloidin by SMTP conjugation, we performed wound-healing scratch assays using the three peptide-phalloidin chimeras. Cytochalasin D was used as a positive control, and vehicle/SFM-only treatment was used as a negative control. Wound-healing assays are migration assays in which the movement of nonreplicating cells into an artificially created cell-free channel is measured over time. Amoeboid cell migration is dependent on repeated actin-driven pseudopodia movements and thus is sensitive to actin inhibition by cytosolic acting phalloidin and cytochalasin D.

In scratch assays, the goal is to observe cell migration into the scratch-created gap area in the absence of cell division. To ensure that cell division was inhibited, cells were cultured for 48 h in serum-free media (SFM) prior to experimentation, which is known to cause cells to enter the G_0_ phase of the cell cycle.^31,32^ Performing experiments in SFM also ensured that peptide activity would not be dampened by potential interactions with FBS.^33,34^

We performed 4 h incubation in the presence of peptide chimeras and then washed the cells with PBS three times prior to conducting the scratch-closing measurements. This approach enabled us to assay only for rapid and nonreversible delivery of phalloidin cargo to the cytosol of cells by the peptides. The control compound cytochalasin D needed to be maintained at the noted concentration throughout the entire incubation time because, unlike the peptides, it is readily washed out of cells and has no effect if incubated for 4 h and washed away. The HT1080 cell monolayers are sensitive to toxic effects; however, none of the peptides or peptide chimeras tested showed any meaningful amount of cytotoxicity, confirming the results described above.

**Figure 4:**
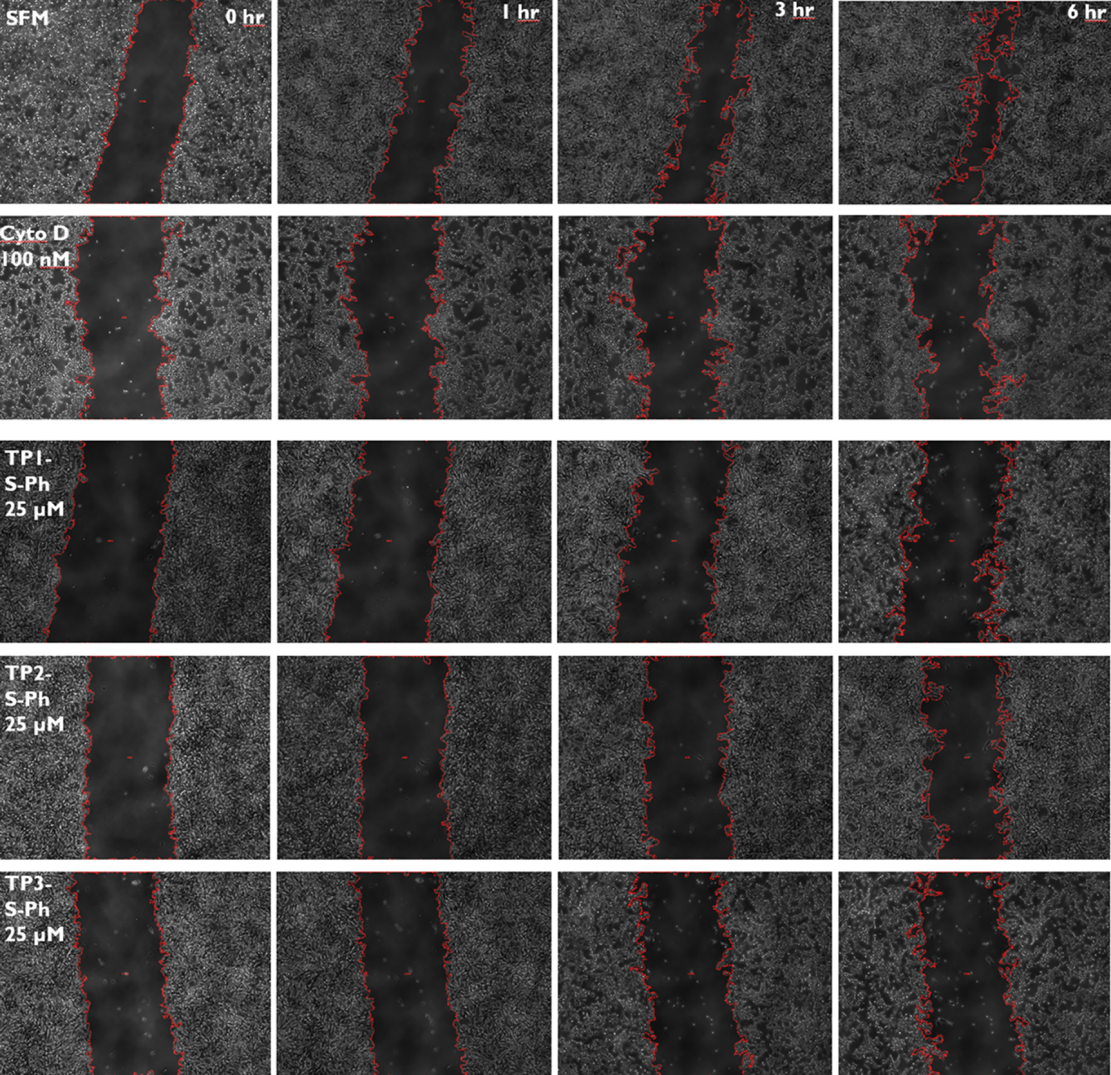
Example scratch assay. For this assay, a total of 30,000 HT1080 cells were seeded into each well of a 96-well cell culture plate in complete media and incubated for 24 h. Cells were washed with DPBS and incubated in serum-free media (SFM) for the next 48 h. Treatment with serum-free media, cytochalasin D, or peptide was done for 4 h at 37 °C. Cells were washed three times with DPBS and then scratched with 10 μL pipet tips on a multichannel pipettor. Cytochalasin D was replaced at the intended concentration, but the peptide was not. Evaluation of wound area was performed by phase contrast microscopic imaging of each well at 4× magnification with a BioTek Cytation 5 cell imaging reader and the BioSpa 8 automated incubator. Here, we show example images from a typical scratch assay taken 0, 1, 3, and 6 h after scratching a channel in the middle of the surface. Red lines show the edge of the wound identified by ImageJ and were used to calculate the total wound area from each image.

In Figure 4, we show representative sets of images produced by using the scratch assay. In these phase contrast images, the wide band down the center of the image is the gap created by scratching a confluent cell population of HT1080 cells with a 10 μL pipet tip, following 4 h of treatment with peptide, drug, or vehicle. The red line surrounding the gap is the trace that ImageJ overlaid onto the image and within the red line is the area of the gap that was measured. In order to appropriately scale these data for accurate comparison, the relative gap area was plotted as the final area over the initial area, with a maximum value of 1.0 at *t* = 0 h and a minimum value of 0 at complete gap closure. In Figure 4, the columns from left to right are 0, 1, 3, and 6 h images. The rows, top to bottom, are SFM, 100 nM continuous cytochalasin D (CD), and 25 μM TP1-S-Ph, TP2-S-Ph, TP3-S-Ph, applied for the first 4 h only. CD, the positive control, inhibited cell movement completely, while SFM treatment, the negative control, yielded maximal cell migration and wound closure. In SFM-treated samples, the channel steadily closes with a halftime of about 3 h. Over the first 6 h, cells treated with 100 nM CD show little to no migration. Migration inhibition by CD is concentration dependent so that treatment with 50 mM CD enabled some migration to take place. At 25 μM, TPx-S-Ph chimeras inhibited cell migration for at least 6 h and the effect of 25 μM TP2-SMCC-Ph was at least as large as the CD control.

**Figure 5:**
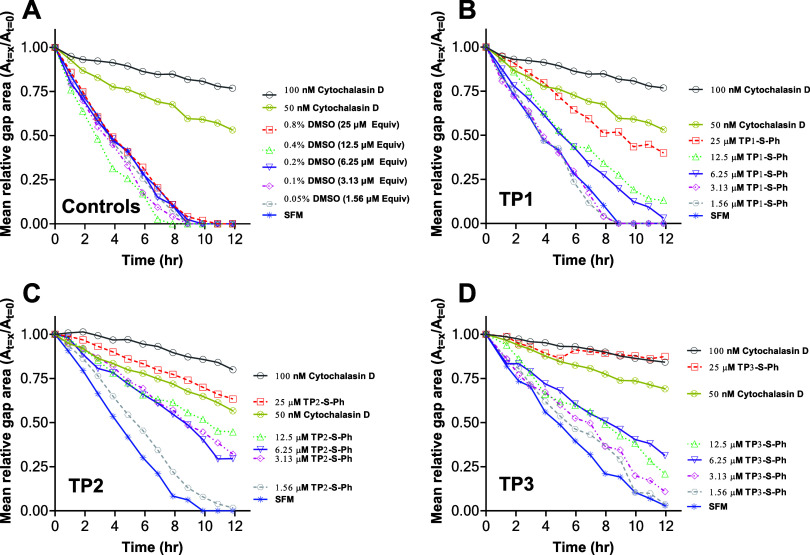
Example wound closure time courses. Wound closure (scratch) assays, like those shown in Figure 4, were quantitated using ImageJ at each time point to measure the total gap area. Each curve is normalized to the time zero wound area for that experiment. (A) Positive and negative controls. Positive control is continuous cytochalasin D. Negative controls include SFM with various DMSO concentrations equivalent to those present in the experiments. (B–D): Example wound closure experiments with the three SMTP-SMCC-Ph conjugates, incubated with cells for 4 h and then washed off, prior to wound creation.

The SMTPs used in this work and their phalloidin chimeras are hydrophobic enough to require solubilization in DMSO to achieve the necessary mM stock concentrations. DMSO is toxic to a wide range of cell lines at 1% v/v and higher, depending on the incubation period and cell line.^35,36^ In these experiments, peptide stocks dissolved in DMSO were diluted into SFM to give a maximum concentration of 0.8% DMSO, equivalent to samples containing 25 μM peptide, which was washed away after the initial 4 h incubation. To verify the lack of DMSO toxicity in HT1080 cells, DMSO concentrations in SFM equivalent to each concentration of TPx-S-Ph were tested in the scratch assays. As shown in Figure 5A, DMSO content up to a concentration equivalent to 25 μM peptide had no significant effect on migration rates compared to SFM-only control. Thus, the differences observed between the migration rates of peptide-treated and vehicle-treated cells are due to the activity of the cargo, phalloidin. For comparison, the effect of the positive control cytochalasin D is shown at 100 and 50 nM continuous concentration.

In Figure 5B–D**,** we show example migration experiments for cytochalasin D at 100 and 50 nM, and for all three TP-S-Ph conjugates, at 25, 12.5, 6.25, 3.13, and 1.56 μM. The data shown are averages of 2–3 technical replicates for one biologically independent experiment. The negative controls of serum-free media and DMSO-equivalents in SFM were aggregated.

For each experiment, we applied linear regression, as shown in Figure 5, to determine the slope of the relative gap area during the most linear portion of time (*t* = 1–8 h). Migration rates were normalized to that of the NT/SFM group, which was always the fastest closing group. Each experiment was repeated in at least 6 biologically independent experiments, and each biologically independent experiment was repeated in 2–3 technical replicates.

**Figure 6:**
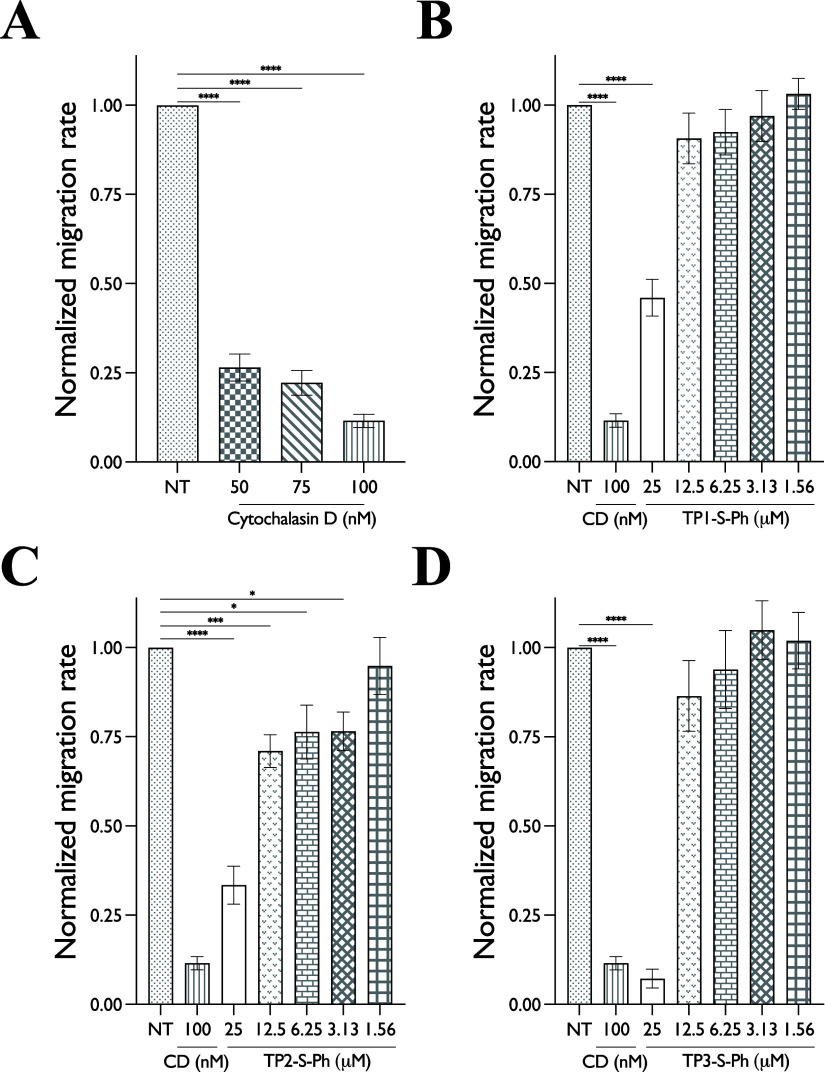
Mean wound closure rates calculated by averaging slopes of at least three independent scratch assays for each experimental condition. NT: No treatment (vehicle only). CD-Cytochalasin D. (A) Continuous Cytochalasin D. (B) TP1-S-Ph incubated for only 4 h before the scratch. (C) TP2-S-Ph incubated for only 4 h before the scratch. (D) TP3-S-Ph incubated for only 4 h before the scratch. All statistical comparisons shown are the results of one-way ANOVA with post hoc comparisons to the effect of no treatment (NT).

If Figure 6, we show averaged wound closure rates, grouped by test compound. Ordinary one-way ANOVA was used to compare vehicle control with each treatment group. Statistically significant pairwise comparisons are indicated in each figure. For continuous CD, all concentrations tested showed significant effects on migration compared to SFM, while DMSO/vehicle controls showed no effect on migration compared to SFM. For TP2-S-Ph, significant effects were noted at 25, 12.5, 6.3, and 3.1 μM peptide-chimera. The effects of TP1 and TP3 chimeras were evident at 25 μM but not at lower concentrations.

## Discussion

All three spontaneous membrane translocating peptides (SMTPs) successfully delivered the cyclic peptide cargo phalloidin to the cell cytosol, where it was able to exert its biological effect for at least 12 h. CD, the positive control, and phalloidin inhibit cell movement, although their mechanisms of action differ slightly. CD binds to the leading edge of the microfilament, while phalloidin binds and stabilizes the monomeric connections in F-actin.^37^ Furthermore, CD is membrane permeant, and thus, it easily enters cells and is easily washed out of cells. For this reason, CD had to be maintained at its experimental concentration during the entire incubation period to have any measurable effect. The peptide-phalloidin chimeras, on the other hand, had measurable effects after only a 4 h incubation, followed by removal of extracellular chimera.

The observation that TP2 is the most efficient peptide tested for delivery of phalloidin correlates with previous findings that TP2 is more active than other members of the SMTP family.^17^ However, why does TP2 effectively deliver cyclic peptide cargo molecules at lower concentrations than TP1 and TP3? The critical translocation motif in the TP2 family of peptides was identified as LRLLR, in positions 5–9, Figure 2.^16,38^ In TP3, the LRLLR motif is changed to LQLLR. However, TP1 contains the full motif. In fact, the only sequence difference between TP1 and TP2 is the L4 in TP1 that is changed to Y4 in TP2. It must follow then that the differences in delivery capacity arise from subtle differences in the physical-chemical properties of the peptides, perhaps affecting solubility, structure, or membrane binding. These observations exemplify our longstanding conclusion that optimization of membrane-active peptides is most efficiently accomplished by synthetic molecular evolution (iterative high-throughput screening) rather than by rational engineering.

This work has demonstrated, for the first time, that SMTPs, namely, TP1, 2, and 3, have the potential to act as platforms for the delivery of cyclic peptides to the cytosol of living cells. The retained ability of phalloidin to bind to F-actin while conjugated to SMTPs may have significant implications for the future design of cyclic peptide drugs that inhibit protein–protein interactions, but releasable cargoes are also easily engineered.^39^ This family of SMTPs is able to deliver membrane-impermeable cyclic peptides to the cell cytosol after a one-time incubation despite the fact that the cargo is not membrane permeant on its own and has a similar molecular weight as the delivery vehicle. However, delivery is much better at 25 μM compared to lower concentrations, indicating that the current 12-residue SMTPs, conjugated to the 7-residue phalloidin, may be near their carrying capacity in terms of size, charge, and/or structural rigidity of the cargo. Nonetheless, the ability of SMTPs to effectively deliver a membrane-impermeable cyclic peptide is remarkable considering that SMTPs were not selected for delivery of this class of cargo, and, in fact, were identified in a screen for delivery of a small fluorescent dye.^16^

The data herein logically support the continued synthetic molecular evolution of the SMTPs for the delivery of cyclic peptide cargos to multiple human cell types. We have shown that synthetic molecular evolution is a powerful approach for the optimization of peptide sequences when the known physical principles do not lead to obvious sequence-function relationships.^16,27,40−43^ For example, the measured differences in delivery efficiency between TP1 and TP2 arise from a single conservative amino acid difference, and the effects of this change are not readily predictable. Future generations of SMTPs, optimized by synthetic molecular evolution, for delivery of specific cyclic peptide cargoes are likely to lead to dramatically improved variants with increased capability for the delivery of bioactive cyclic peptide cargoes into cells.

## Supporting Information Available

Mass spectrometry analysis results (PDF)
