# Supplementary material for: Cytosolic Delivery of Bioactive Cyclic Peptide Cargo by Spontaneous Membrane Translocating Peptides
Source: ACS Omega. 2024 Feb 5;9(7):8179–87. doi: 10.1021/acsomega.3c08701 (PMC10882622; doi:10.1021/acsomega.3c08701)
Supplement: Supplementary file 1 — ao3c08701_si_001.pdf [file ao3c08701_si_001.pdf]

SUPPLEMENTAL DATA FIGURES

Cytosolic delivery of bioactive cyclic peptide cargo by spontaneous membrane translocating peptides

Ryan P. Ferrie[1], Taylor Fuselier[1], William C. Wimley*[1]

[1] Department of Biochemistry and Molecular Biology, Tulane University School of Medicine, New Orleans, LA 70112.

*To whom correspondence should be addressed at wwimley@tulane.edu

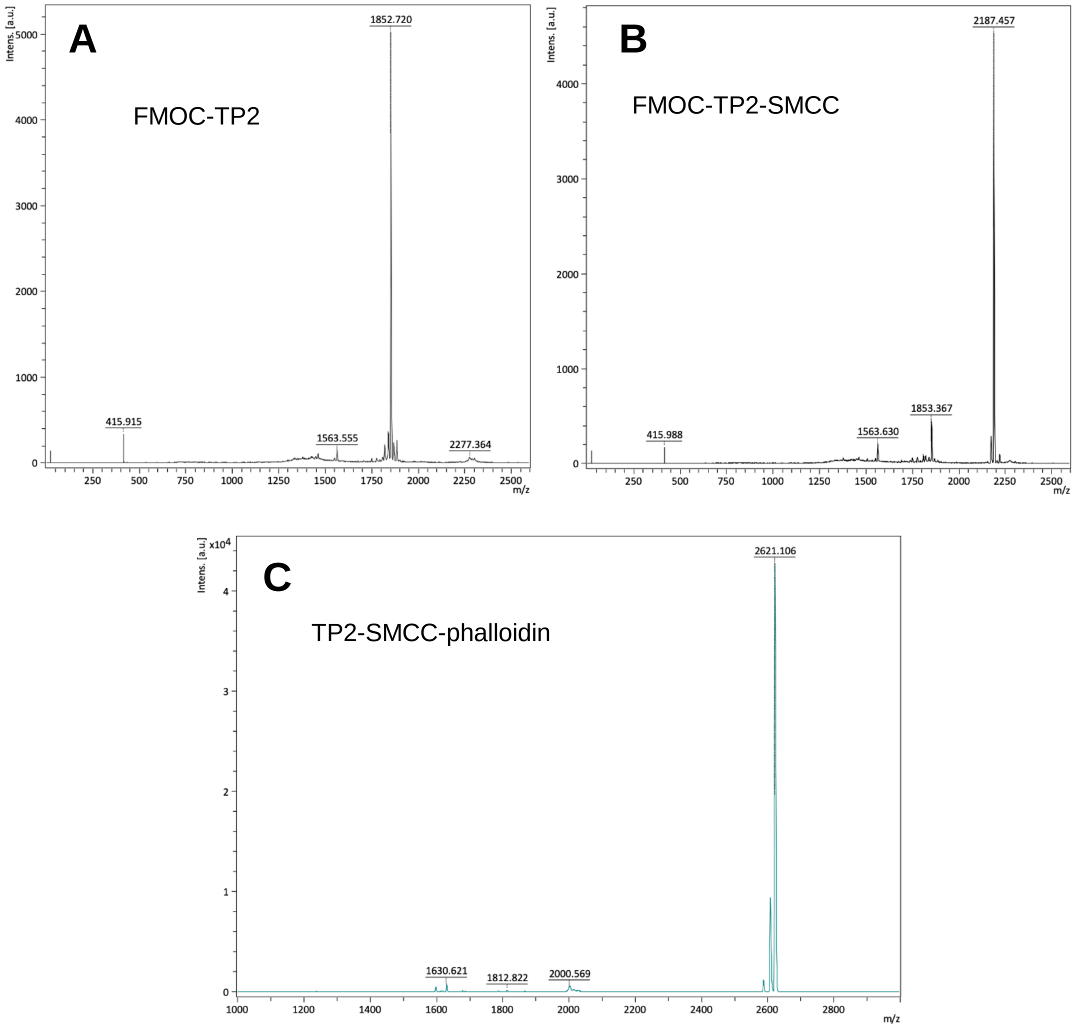

Figure S1. MALDI mass spectrometry analysis of SMTP synthesis and SMTP-SMCC-phalloidin conjugate formation. Spectra shown here are for TP2 which was synthesized on Tentagel-S-NH_2 resin beads, linked by a UV cleavable photolinker. **A.** After solid phase synthesis of FMOC-protected TP2, and cleavage of side chain protecting groups, the resin with peptide attached was thoroughly washed. A small sample was cleaved with UV light for 4 hours and peptide was extracted. The crude product, Fmoc-TP2-amide, shows a dominant peak at the expected mass of 1852 m/z, indicating that synthesis was successful, and the product lacked any significant impurities. **B.** The heterobifunctional crosslinker SMCC was coupled to the peptide on the beads via a maleimide-thiol reaction. After washing away excess reagents, a small sample was cleaved with UV light. The mass spectrometry of this intermediate product, FMOC-TP2-SMCC, showed the expected predominant peak at 2187 m/z. **C.** 7Lys-phalloidin was reacted with FMOC-TM2-SMCC on beads to couple the phalloidin to the TP2-SMCC conjugate via the succinimidyl ester of the SMCC. The peptide FMOC was removed, the resin was washed extensively, and the final product was released from the resin with UV light. The mass spectrometry of the released material showed the expected mass of 2621 for the TP2-SMCC-phalloidin conjugate. Almost no contaminants were detected.

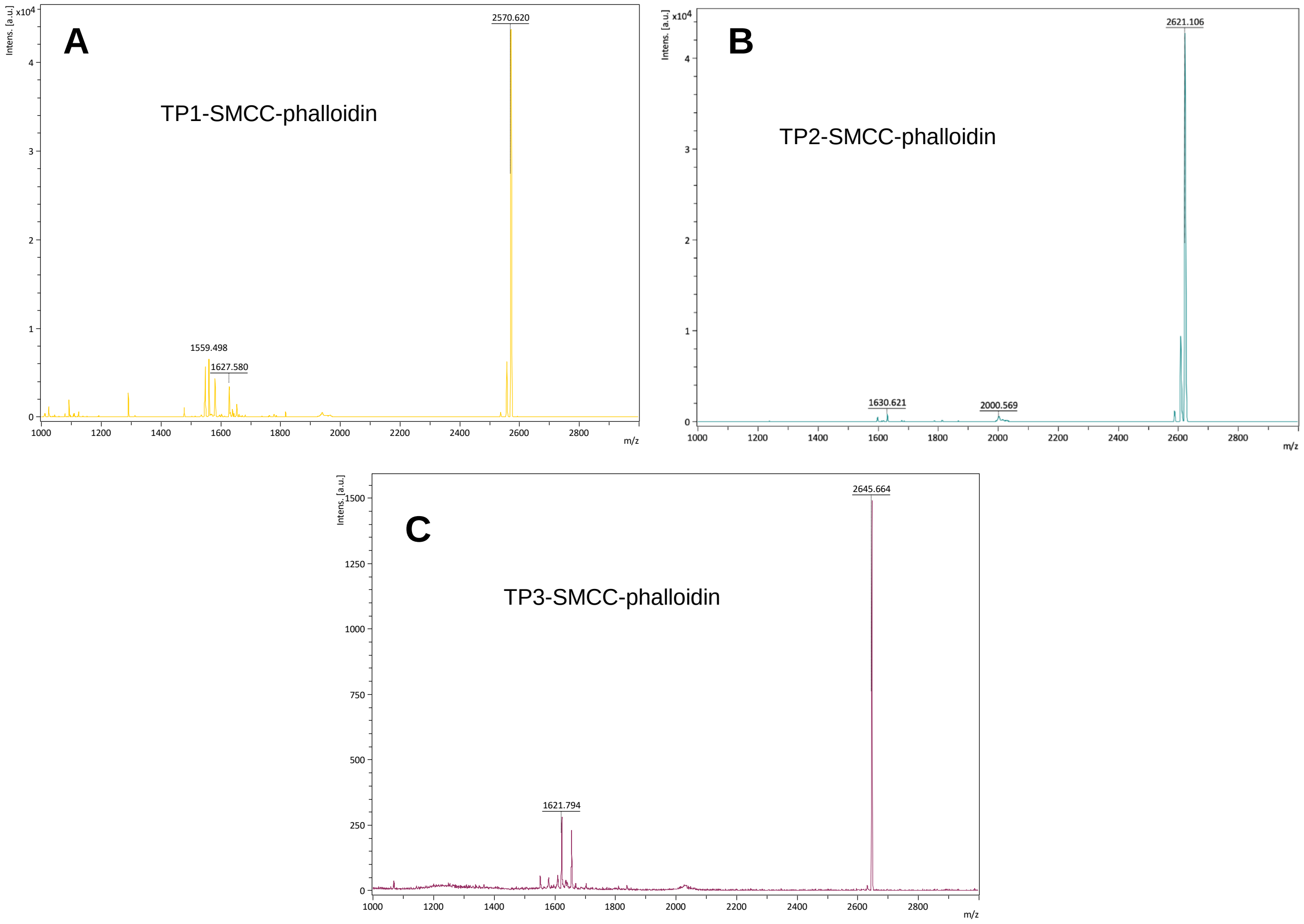

Figure S2. MALDI mass spectrometry analysis of the three SMTP-SMCC-phalloidin conjugates used in this work. Conjugates were formed stepwise on Tentagel-S-NH2 resin using peptides that were linked to the resin by a UV cleavable photolinker. As described in the text, FMOC-protected and sidechain-deprotected peptide on beads were reacted with an excess of SMCC crosslinker which reacted with the cysteine sulfhydryl group via a maleimide moiety. After washing off all reagents, a stoichiometric excess of 7Lys-phalloidin was reacted with the on-bead FMOC-peptide-SMCC to couple the phalloidin to the conjugate via the succinimidyl ester moiety. The FMOC group was removed with piperidine and the bead tethered peptide-SMCC-phalloidin conjugate was washed extensively, prior to release of the conjugate from the beads with UV light. **A.** Mass spectrometry of the released TP1-SMCC-phaloidin showed the expected mass of 2571 for the conjugate. **B.** Mass spectrometry of the released TP2-SMCC-phaloidin showed the expected mass of 2621 for the conjugate. **C.** Mass spectrometry of the released TP3-SMCC-phalloidin showed the expected mass of 2645 for the conjugate.
